# 4-[(*E*)-(5-Chloro-2-hydroxy­benzyl­idene)amino]benzene­sulfonamide

**DOI:** 10.1107/S1600536808040853

**Published:** 2008-12-10

**Authors:** Zahid H. Chohan, Hazoor A. Shad, M. Nawaz Tahir

**Affiliations:** a Department of Chemistry, Bahauddin Zakariya University, Multan-60800, Pakistan; b Department of Physics, University of Sargodha, Sargodha, Pakistan

## Abstract

In the mol­ecule of title compound, C_13_H_11_ClN_2_O_3_S, the aromatic rings are oriented at a dihedral angle of 12.27 (3)°. An intra­molecular O—H⋯N hydrogen bond results in the formation of a planar (mean deviation 0.0083 Å) six-membered ring, which is nearly coplanar with the adjacent ring at a dihedral angle of 2.36 (13)°. In the sulfonamide group, the S atom is 0.457 (3) Å from the plane through the O and N atoms. In the crystal structure, inter­molecular N—H⋯O hydrogen bonds link the mol­ecules.

## Related literature

For general background, see: Chohan (2008 ▶); Chohan & Shad (2008 ▶); Chohan & Supuran (2008 ▶); Nishimori *et al.* (2005 ▶). For related structures, see: Chohan *et al.* (2008*a* ▶,*b* ▶); Shad *et al.* (2008 ▶); Gelbrich *et al.* (2008 ▶). For bond-length data, see: Allen *et al.* (1987 ▶).
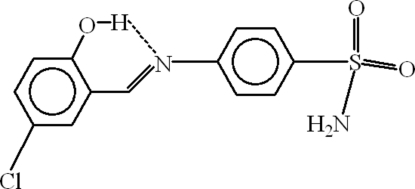


## Experimental

### 

#### Crystal data

C_13_H_11_ClN_2_O_3_S
*M* *_r_* = 310.76
Monoclinic, 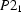

*a* = 6.1936 (9) Å
*b* = 4.6002 (7) Å
*c* = 23.252 (3) Å
β = 95.699 (7)°
*V* = 659.22 (16) Å^3^
*Z* = 2
Mo *K*α radiation
μ = 0.46 mm^−1^
*T* = 296 (2) K
0.25 × 0.18 × 0.15 mm

#### Data collection

Bruker Kappa APEXII CCD diffractometer
Absorption correction: multi-scan (*SADABS*; Bruker, 2005 ▶) *T*_min_ = 0.904, *T*_max_ = 0.935
7850 measured reflections
3172 independent reflections
1842 reflections with *I* > 2σ(*I*)
*R*_int_ = 0.053

#### Refinement

*R*[*F* ^2^ > 2σ(*F* ^2^)] = 0.056
*wR*(*F* ^2^) = 0.132
*S* = 1.02
3172 reflections
193 parameters
4 restraints
H atoms treated by a mixture of independent and constrained refinement
Δρ_max_ = 0.25 e Å^−3^
Δρ_min_ = −0.36 e Å^−3^
Absolute structure: Flack (1983 ▶), 1125 Friedel pairs
Flack parameter: 0.09 (13)

### 

Data collection: *APEX2* (Bruker, 2007 ▶); cell refinement: *SAINT* (Bruker, 2007 ▶); data reduction: *SAINT*; program(s) used to solve structure: *SHELXS97* (Sheldrick, 2008 ▶); program(s) used to refine structure: *SHELXL97* (Sheldrick, 2008 ▶); molecular graphics: *ORTEP-3 for Windows* (Farrugia, 1997 ▶) and *PLATON* (Spek, 2003 ▶); software used to prepare material for publication: *WinGX* (Farrugia, 1999 ▶) and *PLATON*.

## Supplementary Material

Crystal structure: contains datablocks global, I. DOI: 10.1107/S1600536808040853/hk2593sup1.cif

Structure factors: contains datablocks I. DOI: 10.1107/S1600536808040853/hk2593Isup2.hkl

Additional supplementary materials:  crystallographic information; 3D view; checkCIF report

## Figures and Tables

**Table 1:** Hydrogen-bond geometry (Å, °)

*D*—H⋯*A*	*D*—H	H⋯*A*	*D*⋯*A*	*D*—H⋯*A*
O1—H1⋯N1	0.82	1.87	2.603 (5)	148
N2—H21⋯O3^i^	0.87 (4)	2.16 (4)	2.986 (6)	160 (5)

Symmetry code: (i) 
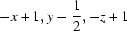
.

**Fig. 1.**
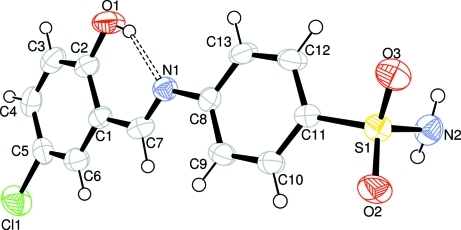
The molecular structure of the title molecule, with the atom-numbering scheme. Displacement ellipsoids are drawn at the 50% probability level. Hydrogen bond is shown as dashed line.

**Fig. 2.**
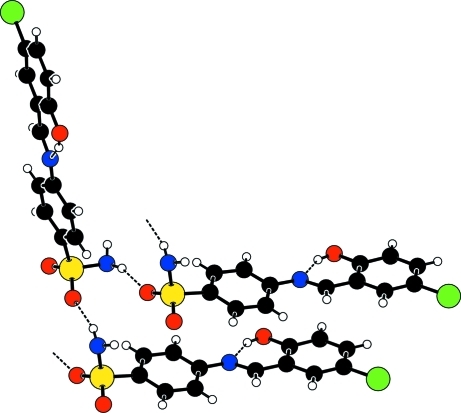
A partial packing diagram of the title compound. Hydrogen bonds are shown as dashed lines.

## supplementary crystallographic information

### Comment

Sulfonamides have gained much attention due to their extensive use in medicine. Many novel sulfonamide derived compounds have been synthesized and reported (Chohan, 2008; Chohan & Shad, 2008; Chohan & Supuran, 2008; Nishimori *et al.*, 2005) that are expected to attack the selective targets. This approach is supportive in controlling undesirable effects and producing distinctive pharmacological and clinical responses. In continuation to synthesize Schiff base ligands of 5-chlorosalicylaldehyde with different sulfonamides (Chohan *et al.*, 2008*a*, 2008*b*; Shad *et al.*, 2008), we have synthesized the title compound having the sulfanilamide, which is also a member of sulfonamides, and reported herein its crystal structure. The crystal structures of the individual moieties of δ-sulfanilamide have also been reported (Gelbrich *et al.*, 2008) .

In the molecule of title compound (Fig 1), the bond lengths (Allen *et al.*, 1987) and angles are within normal ranges. Rings A (C1-C6) and B (C8-C13) are, of course, planar, and the dihedral angle between them is A/B = 12.27 (3)°. The intramolecular O-H···N hydrogen bond (Table 1) results in the formation of a planar six-membered ring C (O1/N1/C1/C2/C7/H1), which is oriented with respect to rings A and B at dihedral angles of A/C = 2.36 (13)° and B/C = 13.22 (13)°. So, rings A and C are also nearly coplanar. In the sulfonamide group, the S1 atom is 0.457 (3) Å away from the plane of (O2/O3/N2).

In the crystal structure, intermolecular N-H···O hydrogen bonds (Table 1) link the molecules (Fig. 2), in which they may be effective in the stabilization of the structure.

### Experimental

For the preparation of the title compound, sulfanilamide (344.4 mg, 2 mmol) in ethanol (20 ml) was mixed with 5-chlorosalicylaldehyde (313.1 mg, 2 mmol) in ethanol (10 ml). The resultant mixture was refluxed for 3 h by monitoring through TLC. During refluxing the solution turned from colorless to bright orange. After completion of reaction, it was cooled to room temperature, filtered and volume reduced to about one-third using rotary evaporator. It was then allowed to stand for 6 d at room temperature. After which, a crystallized product was formed that was filtered, washed with ethanol (2x5 ml), dried and recrystallized in a mixture of methanol/ethanol (1:1) to afford the orange crystals of the title compound (m.p. 469-471 K).

### Refinement

H7 (for CH) and H21, H22 (for NH_2_) atoms were located in difference syntheses and refined isotropically [C-H = 0.97 (4) Å, U_iso_(H) = 0.040 (13) Å^2^; N-H = 0.87 (4) and 0.87 (5) Å; U_iso_(H) = 0.07 (2) and 0.08 (2) Å^2^]. The remaining H atoms were positioned geometrically, with O-H = 0.82 Å (for OH) and C-H = 0.93 Å for aromatic H and constrained to ride on their parent atoms with U_iso_(H) = xU_eq_(C,O), where x = 1.5 for OH H and x = 1.2 for aromatic H atoms.

### Crystal data

**Table:**

C_13_H_11_ClN_2_O_3_S	*F*(000) = 320
*M**_r_* = 310.76	*D*_x_ = 1.566 Mg m^−^^3^
Monoclinic, *P*2_1_	Mo *K*α radiation, λ = 0.71073 Å
Hall symbol: P 2yb	Cell parameters from 1848 reflections
*a* = 6.1936 (9) Å	θ = 0.9–26.4°
*b* = 4.6002 (7) Å	µ = 0.46 mm^−^^1^
*c* = 23.252 (3) Å	*T* = 296 K
β = 95.699 (7)°	Prism, orange
*V* = 659.22 (16) Å^3^	0.25 × 0.18 × 0.15 mm
*Z* = 2	

### Data collection

**Table:**

Bruker Kappa APEXII CCD diffractometer	3172 independent reflections
Radiation source: fine-focus sealed tube	1842 reflections with *I* > 2σ(*I*)
graphite	*R*_int_ = 0.053
Detector resolution: 7.6 pixels mm^-1^	θ_max_ = 28.5°, θ_min_ = 0.9°
ω scans	*h* = −8→8
Absorption correction: multi-scan (*SADABS*; Bruker, 2005)	*k* = −6→6
*T*_min_ = 0.904, *T*_max_ = 0.935	*l* = −31→29
7850 measured reflections	

### Refinement

**Table:**

Refinement on *F*^2^	Secondary atom site location: difference Fourier map
Least-squares matrix: full	Hydrogen site location: inferred from neighbouring sites
*R*[*F*^2^ > 2σ(*F*^2^)] = 0.056	H atoms treated by a mixture of independent and constrained refinement
*wR*(*F*^2^) = 0.132	*w* = 1/[σ^2^(*F*_o_^2^) + (0.0408*P*)^2^ + 0.3674*P*] where *P* = (*F*_o_^2^ + 2*F*_c_^2^)/3
*S* = 1.02	(Δ/σ)_max_ = 0.001
3172 reflections	Δρ_max_ = 0.25 e Å^−^^3^
193 parameters	Δρ_min_ = −0.36 e Å^−^^3^
4 restraints	Absolute structure: Flack (1983), 1125 Friedel pairs
Primary atom site location: structure-invariant direct methods	Flack parameter: 0.09 (13)

### Special details

**Table:**

Geometry. All esds (except the esd in the dihedral angle between two l.s. planes) are estimated using the full covariance matrix. The cell esds are taken into account individually in the estimation of esds in distances, angles and torsion angles; correlations between esds in cell parameters are only used when they are defined by crystal symmetry. An approximate (isotropic) treatment of cell esds is used for estimating esds involving l.s. planes.
Refinement. Refinement of F^2^ against ALL reflections. The weighted R-factor wR and goodness of fit S are based on F^2^, conventional R-factors R are based on F, with F set to zero for negative F^2^. The threshold expression of F^2^ > 2sigma(F^2^) is used only for calculating R-factors(gt) etc. and is not relevant to the choice of reflections for refinement. R-factors based on F^2^ are statistically about twice as large as those based on F, and R- factors based on ALL data will be even larger.

### Fractional atomic coordinates and isotropic or equivalent isotropic displacement parameters (Å^2^)

**Table:**

	*x*	*y*	*z*	*U*_iso_*/*U*_eq_	
S1	0.76662 (18)	1.3639 (2)	0.57243 (4)	0.0351 (3)	
Cl1	0.7848 (2)	−0.1726 (4)	0.96987 (6)	0.0659 (4)	
O1	0.1496 (5)	0.3015 (9)	0.79117 (14)	0.0554 (10)	
H1	0.2062	0.4241	0.7719	0.083*	
O2	0.9594 (5)	1.5110 (7)	0.59629 (14)	0.0498 (9)	
O3	0.5902 (5)	1.5306 (7)	0.54481 (14)	0.0472 (9)	
N1	0.4614 (6)	0.6117 (9)	0.75542 (16)	0.0365 (9)	
N2	0.8345 (8)	1.1435 (9)	0.52410 (19)	0.0431 (11)	
H21	0.726 (6)	1.068 (12)	0.5029 (19)	0.07 (2)*	
H22	0.929 (8)	1.022 (12)	0.541 (2)	0.08 (2)*	
C1	0.5139 (7)	0.2913 (9)	0.83587 (19)	0.0344 (11)	
C2	0.2983 (8)	0.1959 (11)	0.8319 (2)	0.0394 (12)	
C3	0.2389 (8)	−0.0151 (12)	0.8696 (2)	0.0490 (13)	
H3	0.0970	−0.0837	0.8659	0.059*	
C4	0.3850 (8)	−0.1254 (15)	0.91213 (19)	0.0501 (12)	
H4	0.3418	−0.2638	0.9378	0.060*	
C5	0.5997 (8)	−0.0272 (11)	0.91650 (19)	0.0430 (12)	
C6	0.6617 (8)	0.1767 (11)	0.8793 (2)	0.0420 (12)	
H6	0.8048	0.2409	0.8827	0.050*	
C7	0.5892 (8)	0.4971 (11)	0.7953 (2)	0.0391 (12)	
H7	0.742 (7)	0.545 (10)	0.8017 (17)	0.040 (13)*	
C8	0.5381 (7)	0.8040 (10)	0.71469 (17)	0.0335 (11)	
C9	0.7445 (7)	0.9293 (10)	0.72084 (19)	0.0425 (13)	
H9	0.8379	0.8935	0.7539	0.051*	
C10	0.8097 (8)	1.1053 (10)	0.67816 (19)	0.0385 (12)	
H10	0.9476	1.1875	0.6824	0.046*	
C11	0.6728 (7)	1.1609 (9)	0.62919 (18)	0.0312 (10)	
C12	0.4655 (7)	1.0440 (11)	0.6241 (2)	0.0422 (12)	
H12	0.3702	1.0855	0.5916	0.051*	
C13	0.4006 (7)	0.8677 (14)	0.66654 (18)	0.0411 (11)	
H13	0.2612	0.7903	0.6627	0.049*	

### Atomic displacement parameters (Å^2^)

**Table:**

	*U*^11^	*U*^22^	*U*^33^	*U*^12^	*U*^13^	*U*^23^
S1	0.0380 (7)	0.0288 (5)	0.0380 (6)	−0.0026 (6)	0.0009 (5)	−0.0002 (6)
Cl1	0.0711 (9)	0.0698 (10)	0.0545 (8)	0.0074 (9)	−0.0056 (7)	0.0153 (8)
O1	0.0333 (18)	0.065 (3)	0.066 (2)	−0.0079 (19)	−0.0044 (17)	0.012 (2)
O2	0.049 (2)	0.048 (2)	0.051 (2)	−0.0232 (18)	−0.0069 (17)	0.0015 (17)
O3	0.050 (2)	0.0369 (19)	0.053 (2)	0.0081 (17)	−0.0039 (17)	0.0064 (16)
N1	0.035 (2)	0.037 (2)	0.038 (2)	−0.0014 (18)	0.0037 (19)	0.0012 (18)
N2	0.047 (3)	0.040 (3)	0.043 (3)	−0.003 (2)	0.011 (2)	−0.010 (2)
C1	0.031 (3)	0.033 (3)	0.040 (3)	0.002 (2)	0.007 (2)	−0.003 (2)
C2	0.032 (3)	0.040 (3)	0.046 (3)	−0.002 (2)	0.007 (2)	−0.003 (2)
C3	0.042 (3)	0.052 (3)	0.056 (3)	−0.007 (3)	0.018 (3)	−0.002 (3)
C4	0.060 (3)	0.052 (3)	0.042 (3)	−0.006 (4)	0.021 (2)	0.000 (3)
C5	0.054 (3)	0.044 (3)	0.031 (3)	0.007 (3)	0.005 (2)	−0.001 (2)
C6	0.037 (3)	0.042 (3)	0.047 (3)	0.001 (2)	0.003 (2)	−0.004 (2)
C7	0.028 (3)	0.038 (3)	0.052 (3)	−0.004 (2)	0.005 (3)	−0.002 (2)
C8	0.033 (3)	0.035 (3)	0.034 (2)	0.003 (2)	0.008 (2)	−0.003 (2)
C9	0.034 (3)	0.054 (4)	0.038 (3)	−0.003 (2)	−0.006 (2)	0.007 (2)
C10	0.030 (3)	0.045 (3)	0.039 (3)	−0.006 (2)	−0.002 (2)	0.002 (2)
C11	0.028 (3)	0.030 (2)	0.036 (3)	0.000 (2)	0.001 (2)	−0.002 (2)
C12	0.029 (3)	0.049 (3)	0.046 (3)	0.000 (2)	−0.008 (2)	0.008 (2)
C13	0.024 (2)	0.049 (3)	0.050 (3)	−0.003 (3)	0.005 (2)	0.008 (3)

### Geometric parameters (Å, °)

**Table:**

Cl1—C5	1.738 (5)	C6—C5	1.358 (7)
S1—O2	1.435 (3)	C6—H6	0.9300
S1—O3	1.434 (3)	C7—N1	1.272 (6)
S1—N2	1.600 (4)	C7—C1	1.446 (6)
S1—C11	1.762 (5)	C7—H7	0.97 (4)
O1—H1	0.8200	C8—N1	1.412 (5)
N2—H21	0.87 (4)	C9—C8	1.397 (6)
N2—H22	0.87 (5)	C9—C10	1.372 (6)
C2—O1	1.344 (5)	C9—H9	0.9300
C2—C3	1.381 (7)	C10—H10	0.9300
C2—C1	1.400 (6)	C11—C10	1.375 (6)
C3—C4	1.370 (7)	C11—C12	1.386 (6)
C3—H3	0.9300	C12—H12	0.9300
C4—H4	0.9300	C13—C8	1.370 (6)
C5—C4	1.398 (7)	C13—C12	1.369 (7)
C6—C1	1.397 (6)	C13—H13	0.9300
			
O2—S1—N2	107.7 (2)	C6—C5—C4	120.3 (5)
O2—S1—C11	106.48 (19)	C1—C6—H6	119.5
O3—S1—O2	119.3 (2)	C5—C6—C1	121.0 (5)
O3—S1—N2	105.4 (2)	C5—C6—H6	119.5
O3—S1—C11	109.0 (2)	N1—C7—C1	121.9 (4)
N2—S1—C11	108.6 (2)	N1—C7—H7	123 (3)
C2—O1—H1	109.5	C1—C7—H7	115 (3)
C7—N1—C8	121.4 (4)	C9—C8—N1	123.7 (4)
S1—N2—H21	114 (4)	C13—C8—C9	118.9 (4)
S1—N2—H22	108 (4)	C13—C8—N1	117.3 (4)
H21—N2—H22	117 (6)	C8—C9—H9	119.9
C2—C1—C7	122.0 (4)	C10—C9—C8	120.1 (4)
C6—C1—C2	118.8 (4)	C10—C9—H9	119.9
C6—C1—C7	119.2 (4)	C9—C10—C11	120.5 (4)
O1—C2—C1	121.0 (4)	C9—C10—H10	119.8
O1—C2—C3	119.6 (5)	C11—C10—H10	119.8
C3—C2—C1	119.4 (5)	C10—C11—C12	119.3 (4)
C2—C3—H3	119.3	C10—C11—S1	119.8 (4)
C4—C3—C2	121.3 (5)	C12—C11—S1	120.8 (3)
C4—C3—H3	119.3	C11—C12—H12	119.9
C3—C4—C5	119.2 (5)	C13—C12—C11	120.2 (4)
C3—C4—H4	120.4	C13—C12—H12	119.9
C5—C4—H4	120.4	C8—C13—H13	119.6
C4—C5—Cl1	118.9 (4)	C12—C13—C8	120.9 (4)
C6—C5—Cl1	120.9 (4)	C12—C13—H13	119.6
			
O2—S1—C11—C10	−17.2 (4)	C1—C6—C5—C4	−0.3 (7)
O2—S1—C11—C12	165.9 (4)	C1—C6—C5—Cl1	179.2 (4)
O3—S1—C11—C10	−147.1 (4)	N1—C7—C1—C6	−179.0 (5)
O3—S1—C11—C12	36.0 (4)	N1—C7—C1—C2	3.1 (7)
N2—S1—C11—C10	98.5 (4)	C1—C7—N1—C8	−177.6 (4)
N2—S1—C11—C12	−78.4 (4)	C9—C8—N1—C7	−13.3 (7)
O1—C2—C1—C6	179.5 (4)	C13—C8—N1—C7	166.4 (5)
O1—C2—C1—C7	−2.6 (7)	C10—C9—C8—N1	177.4 (4)
C3—C2—C1—C6	−2.1 (7)	C10—C9—C8—C13	−2.2 (7)
C3—C2—C1—C7	175.8 (4)	C8—C9—C10—C11	0.3 (7)
O1—C2—C3—C4	−179.1 (5)	S1—C11—C10—C9	−175.1 (4)
C1—C2—C3—C4	2.4 (8)	C12—C11—C10—C9	1.8 (7)
C2—C3—C4—C5	−1.6 (8)	S1—C11—C12—C13	174.8 (4)
C6—C5—C4—C3	0.5 (8)	C10—C11—C12—C13	−2.0 (7)
Cl1—C5—C4—C3	−179.0 (4)	C12—C13—C8—N1	−177.6 (5)
C5—C6—C1—C2	1.1 (7)	C12—C13—C8—C9	2.0 (8)
C5—C6—C1—C7	−176.9 (4)	C8—C13—C12—C11	0.1 (8)

### Hydrogen-bond geometry (Å, °)

**Table:**

*D*—H···*A*	*D*—H	H···*A*	*D*···*A*	*D*—H···*A*
O1—H1···N1	0.82	1.87	2.603 (5)	148
N2—H21···O3^i^	0.87 (4)	2.16 (4)	2.986 (6)	160 (5)

Symmetry codes: (i) −*x*+1, *y*−1/2, −*z*+1.
